# Supplementary material for: Identification of photoperiod-induced specific miRNAs in the adrenal glands of Sunite sheep (Ovis aries)
Source: Front Vet Sci. 2022 Jul 22;9:888207. doi: 10.3389/fvets.2022.888207 (PMC9354845; doi:10.3389/fvets.2022.888207)
Supplement: Supplementary file 1 [file Data_Sheet_1.ZIP › Supplementary file/miRDeep2 file.zip!/miRDeep2 file/Know_Structure/SP42A3/oar-let-7d.pdf]

miRBase precursor	: oar-let-7d
Total read count	: 23298
oar-let-7d read count	: 21196
remaining reads	: 2102

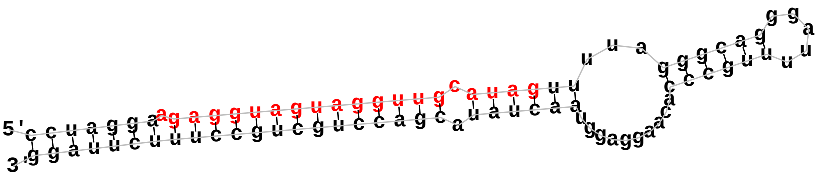

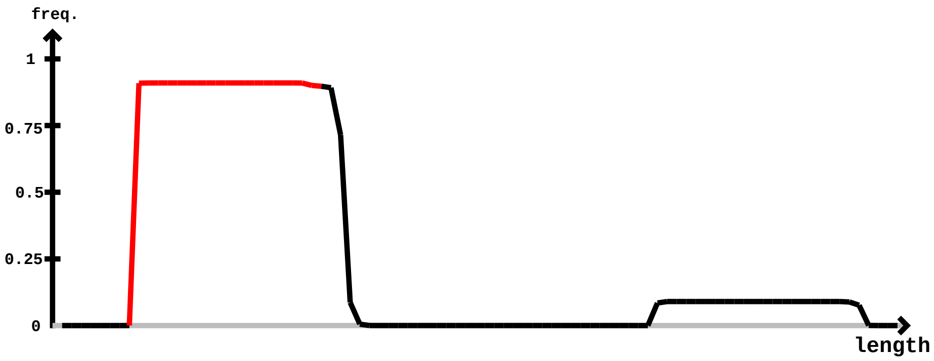


```
                oar-let-7d
5'- ccuaggaagagguaguagguugcauaguuuuagggcagggauuuugcccacaaggagguaacuauacgaccugcugccuuucuuagg   -3'    exp
    (((((((.((((((((((((((.((((((...((((((.....))))))..........)))))).)))))))))))))))))))))   reads      mm         sample
    ......aagagguaguagguugcauagu...........................................................   4          0          seq
    ......aagagguaguagguugcauaguu..........................................................   1          0          seq
    .......agagguaguagguugca...............................................................   6          0          seq
    .......agagguaguagguuAcau..............................................................   1          1          seq
    .......agagguaguagguugcau..............................................................   211        0          seq
    .......agagguaguagguugcaua.............................................................   73         0          seq
    .......agagguaguagguugcauag............................................................   125        0          seq
    .......agagguaguagguugcauagu...........................................................   4124       0          seq
    .......agagguaguagguugcauaguu..........................................................   14618      0          seq
    .......agagguaguagguugcauaguuu.........................................................   1886       0          seq
    .......agagguaguagguugcauaguuuu........................................................   124        0          seq
    .......agagguaguagguugcauaguuuua.......................................................   2          0          seq
    ........gagguaguagguugcauagu...........................................................   3          0          seq
    ........gagguaguagguugcauaguu..........................................................   15         0          seq
    ........gagguaguagguugcauaguuu.........................................................   2          0          seq
    .........agguaguagguugcauaguuu.........................................................   1          0          seq
    .............................uuagggcagggauuuugcccacaaggaggu............................   2          0          seq
    .............................................................cuauacgaccugcugccuu.......   1          0          seq
    .............................................................cuauacgaccugcugccuuu......   36         0          seq
    .............................................................cuauacgaccugcugccuuuc.....   266        0          seq
    .............................................................cuauacgaccugcugccuuucu....   1658       0          seq
    .............................................................cuauacgaccugcugccuuucuu...   11         0          seq
    ..............................................................uauacgaccugcugccuuu......   4          0          seq
    ..............................................................uauacgaccugcugccuuuc.....   15         0          seq
    ..............................................................uauacgaccugcugccuuucu....   108        0          seq
    ...............................................................auacgaccugcugccuuuc.....   1          0          seq
```
